# Identification of Locally Advanced Rectal Cancer with Low Risk of Local Recurrence

**DOI:** 10.1371/journal.pone.0117141

**Published:** 2015-01-28

**Authors:** Qiao-Xuan Wang, Shao-Hua Li, Xu Zhang, Lan Xie, Pei-Qiang Cai, Xin An, Zhi-Zhong Pan, Pei-Rong Ding

**Affiliations:** 1 State Key Laboratory of Oncology in South China; Collaborative Innovation Center for Cancer Medicine, Guangzhou, P R. China, Sun Yat-sen University Cancer Center, Guangzhou, P. R. China; 2 Department of Radiation Oncology, Sun Yat-sen University Cancer Center, Guangzhou, P. R. China; 3 Department of Hepatobiliary Oncology, Sun Yat-sen University Cancer Center, Guangzhou, P. R. China; 4 Department of Thoracic Surgery, Sun Yat-sen University Cancer Center, Guangzhou, P. R. China; 5 Department of Anesthesiology, Sun Yat-sen University Cancer Center, Guangzhou, P. R. China; 6 Department of Medical Imaging & Interventional Radiology, Sun Yat-sen University Cancer Center, Guangzhou, P. R. China; 7 Department of Medical Oncology, Sun Yat-sen University Cancer Center, Guangzhou, P. R. China; 8 Department of Colorectal Surgery, Sun Yat-sen University Cancer Center, Guangzhou, P. R. China; University of North Carolina School of Medicine, UNITED STATES

## Abstract

**Background:**

The routine application of neoadjuvant chemoradiotherapy for T3N0 rectal cancer remains controversial. The aim of this study was to use clinical, Magnetic resonance imaging, and pathological parameters to identify a subgroup of patients with low risk of local recurrence who might be precluded from neoadjuvant chemoradiotherapy.

**Methods:**

We retrospectively reviewed a prospectively maintained database of consecutive rectal cancer patients who underwent curative resection. 166 pathologic confirmed T3N0 rectal cancer patients with tumor located 5–12cm above the anal verge and preoperative circumferential resection margin>1mm were included in analysis. The primary outcomes measured were3- and 5-year local recurrence rates.

**Results:**

Local recurrence was demonstrated during follow-up in 5 patients; the actuarial overall 3- and 5-year local recurrence rates were 2.5% and 3.4%, respectively. Inadequate sampling of lymph nodes (≤12) was associated with higher local recurrence (P = 0.03) in this group of patients.

**Conclusion:**

For upper and middle T3N0 rectal cancer with preoperative circumferential resection margin>1mm, local recurrence rate after total mesorectal excision is low and surgery alone may be enough for this group of patients.

## Introduction

The current standard therapy for locally advanced rectal cancer is neoadjuvant chemoradiotherapy (CRT) followed by total mesorectal excision (TME) [1]. However, several studies have reported that local recurrence can be well controlled at a relatively low level (4–8%) by surgery alone in patients with T3N0 rectal cancer, suggesting that neoadjuvant CRT might not be necessary for these patients [2–5]. However, not all patients with T3N0 can be spared from neoadjuvant CRT. Risk of local recurrence is also significantly associated with several other factors like location of tumor [6] and circumferential resection margin (CRM) status [7, 8], which should also be taken into account when determine the necessity of neoadjuvant CRT. The European Society for Medical Oncology (ESMO) guideline for treatment of rectal cancer recommends a flexible strategy on application of neoadjuvant therapy basing on clinical staging, location of tumor, and risk of CRM involvement [9], although the evidence is still limited.

Since neoadjuvant CRT only reduces risk of local recurrence but not distant metastases [10], and inevitably results in short-term and long-term toxicities [11], some studies questioned the strategy of routine application of neoadjuvant CRT to patients with T3N0 rectal cancer, and proposed that only those at high risk of local recurrence should be treated with neoadjuvant CRT [2, 12].

In this study, we used clinical, Magnetic resonance imaging (MRI), and pathological parameters to identify patients with low risk of local recurrence who might be precluded from neoadjuvant CRT.

## Materials and Methods

### Patient Selection

Patients were identified from a prospective maintained database in the Sun Yat-sen University Cancer Center from January 2005 to December 2010. The study was performed following approval by the ethic committee of Sun Yat-Sen University Cancer Center. We were replied that it’s not necessary to get signatures of patients’ informed consent forms according to the current Chinese medical regulations. The process of the whole study is retrospective, non-invasive, and without any patients’ benefit hurt. Ethics committees approved this consent procedure. The inclusion criteria were as follows: (1) pathologically confirmed T3N0 rectal adenocarcinoma; (2) tumor located 5–12cm above the anal verge; (3) underwent complete curative resection according to the principles of TME; (4) preoperative CRM >1mm in MRI; The exclusion criteria were as follows: (1) patients received neoadjuvant therapy; (2) existence of distant metastases; (3) history of a second primary malignancy. Follow-up, primarily obtained from the institution database, was updated by clinical chart review, physician records, patient correspondence, and telephone interviews.

### Treatment Scheme

Preoperative evaluation included history/physical, rigid proctoscopy, colonoscopy, chest x-ray or computed tomography (CT) scan, CT scan of the abdomen, endorectal ultrasound, MRI of the pelvis, and serum carcinoembryonic antigen (CEA) levels. All patients received radical anterior resection according to the principles of TME. All patients were followed at 3-month intervals during the first 2 years after surgery and every 6-month thereafter for an additional period of 3 years. Colonoscopy was done one year after surgery. Ultrasonography of the liver was carried out every 3 months. CT scans of chest, abdomen, and pelvis were performed every year for 5 years. Other investigations were performed when clinically indicated during follow-up.

### Evaluation of CRM Status on MRI

CRM involvement was evaluated on pre-operative MRI [13]. The evaluation was done retrospectively by one radiologist who was blinded to the pathology staging. Only patients with mesorectal fascia>1mm were included in this study.

### Statistics

Characteristics were described in terms of frequency for the categorical variables and medians for non-normally distributed continuous. Significance was set at P< 0.05. Primary study end points included 3- and 5-year local recurrence rates, relapse free survival (RFS), and disease-specific survival (DSS). Local recurrence was defined as recurrence in the pelvis whether newly diagnosed distant metastases were present or not. Local recurrence and patient survival rates were calculated using the Kaplan-Meier method (with log-rank test). Statistical analyses were performed using the Statistical Package for the Social Sciences program (SPSS Inc. Chicago, IL, version 15.0 for Windows).

## Results

### Demographic and Clinical Pathologic Characteristics

A total of 166 patients were included. Patient and tumor characteristics were listed in Table 1. The median age at diagnosis was 60 years (range, 27 to 86 years). The median number of dissected lymph nodes was 14 (range, 2 to 34). Median distance from the surgical margin was 3.0 cm (range, 1.0 to 7.0 cm).

**Table 1:** Demographic and clinicalpathologic characteristics.

Variable	
Total No. of patients	166
Gender, no. (%)	
Male	111 (66.9)
Female	55 (33.1)
Age at diagnosis, median (range), y	60 (27~86)
Distance to anal verge, median(range), cm	7 (5~12)
Grade, no. (%)	(n = 163)
Ⅰ	4 (2.5)
Ⅱ	147 (90.2)
Ⅲ	12 (7.3)
Mucin production, no. (%)	
Present	6 (3.6)
Absent	160 (96.4)
Preoperative serum CEA level, no. (%)	(n = 164)
≤5 g/ml	112 (68.3)
>5 g/ml	52 (31.7)
Distance from the distal margin, no. (%)	(n = 151)
≤ 2cm	40 (26.5)
>2cm	111 (73.5)
No. of lymph nodes retrieved, no. (%)	
≤12	60 (36.1)
>12	106 (63.9)
Adjuvant chemotherapy, no. (%)	
No	71 (42.8)
Yes	95 (57.2)

### Follow-up and Survival

**Figure 1:**
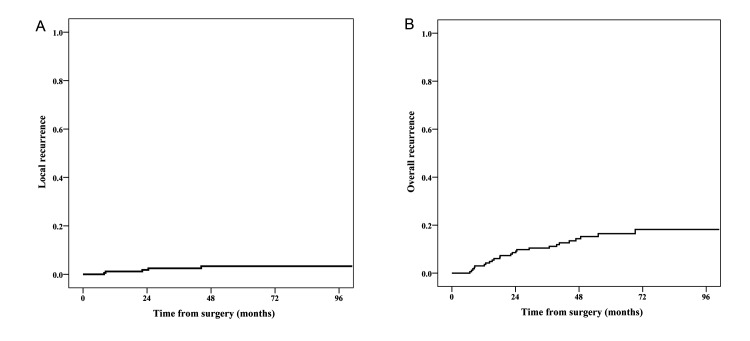
Local and overall recurrence rates in patients with low-risk locally advanced rectal cancer. (A) Cumulative local recurrence rate. (B) Cumulative overall recurrence rate.

The median follow-up was 55.4 months (range, 11.7 to 100.9 months). Local recurrence was demonstrated during follow-up in 5 patients; two of them also had distant metastases detected at the same time. The actuarial overall 3- and 5-year local recurrence rates were 2.5% and 3.4%, respectively (Fig. 1A). Twenty-two patients developed distant metastases, mostly lung metastases or liver metastases (Table 2). The actuarial 3- and 5-year overall recurrence rates were 10.5% and 16.5%, respectively (Fig. 1B). In total, 16 patients died because of recurrent disease; 1 patient died from other reason. 3- and 5-year DSS were 95.6% and 90.2%, respectively.

**Table 2:** Survival and recurrence.

Outcome	
Follow-up, months	
Median	55.4
Range	11.7~100.9
Status, no. (%)	
Recurrence	25 (15.1)
No evidence detected	141 (84.9)
3-year RFS, %	89.5
5-year RFS, %	83.5
Recurrence site, no.	
Local recurrence	5
Distant metastases	22
Lung	11
Liver	6
Brain	2
Peritoneum	1
Bone	1
Lung+liver+peritoneum	1

Abbreviations: RFS, relapse free survival.

### Prognostic Factors

Details of these five patients who developed local recurrence were provided in Table 3. Most recurrence occurred in male patients (4/5), with number of lymph nodes retrieved ≤12 (4/5). Local recurrence and distant metastasis rates were not significantly different in gender (male vs. female), preoperative CEA levels (≤5ng/ml vs. >5mg/ml), histology (grade I vs. grade II vs. grade III), and mucin production (mucinous adenocarcinoma vs. adenocarcinoma not otherwise specified). There was also no significant difference between patients with and without adjuvant chemotherapy in local recurrence rates (P = 0.91), RFS (P = 0.88), and DSS (P = 0.78).

**Table 3:** Details of patients with local recurrence.

No.	Age	Gender	Tumor dimension (cm)	Distance to anal verge (cm)	Distance from the distal margin (cm)	Preoperative CEA level (ng/ml)	Histology	Number of lymph node retrieved	Distant metastasis detected at the same time	RFS (months)
1	72	Male	3.5	6.0	2	3.21	Grade II	15	No	7.9
2	74	Male	2	10.0	2	3.22	Grade II	3	Yes	24.5
3	73	Male	5.5	5.0	2	3.79	Grade I	8	No	8.5
4	41	Female	4	10.0	3	5.15	Grade III	2	Yes	44.3
5	75	Male	3	12.0	7	6.55	Grade II	12	No	22.2

Abbreviations: CEA, serum carcinoembryonic antigen; RFS, relapse free survival.

**Figure 2:**
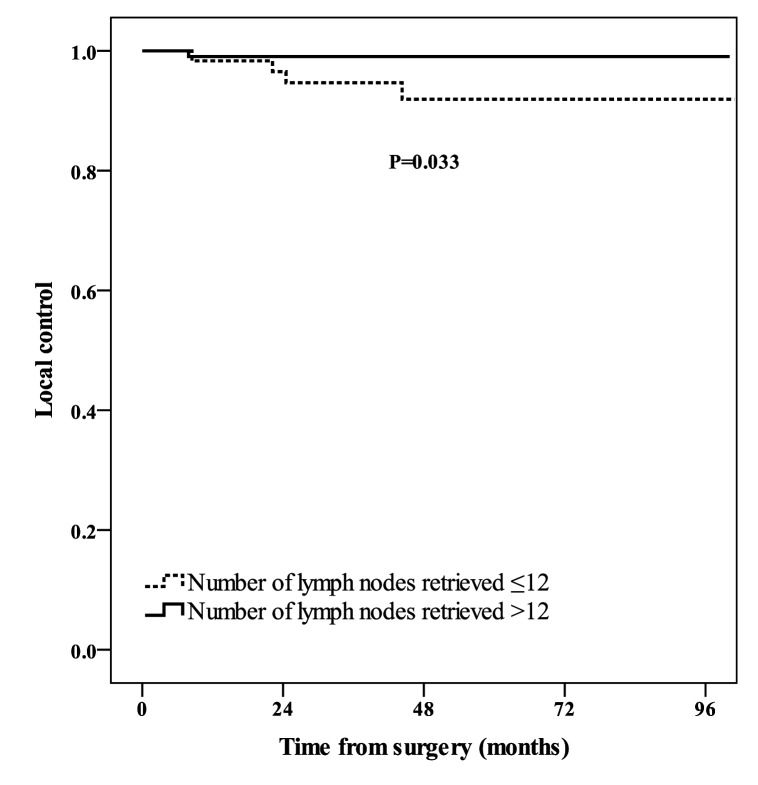
Local control of patients with number of lymph nodes retrieved≤12 (dashed line), and number of lymph nodes retrieved>12 (solid line).

**Figure 3:**
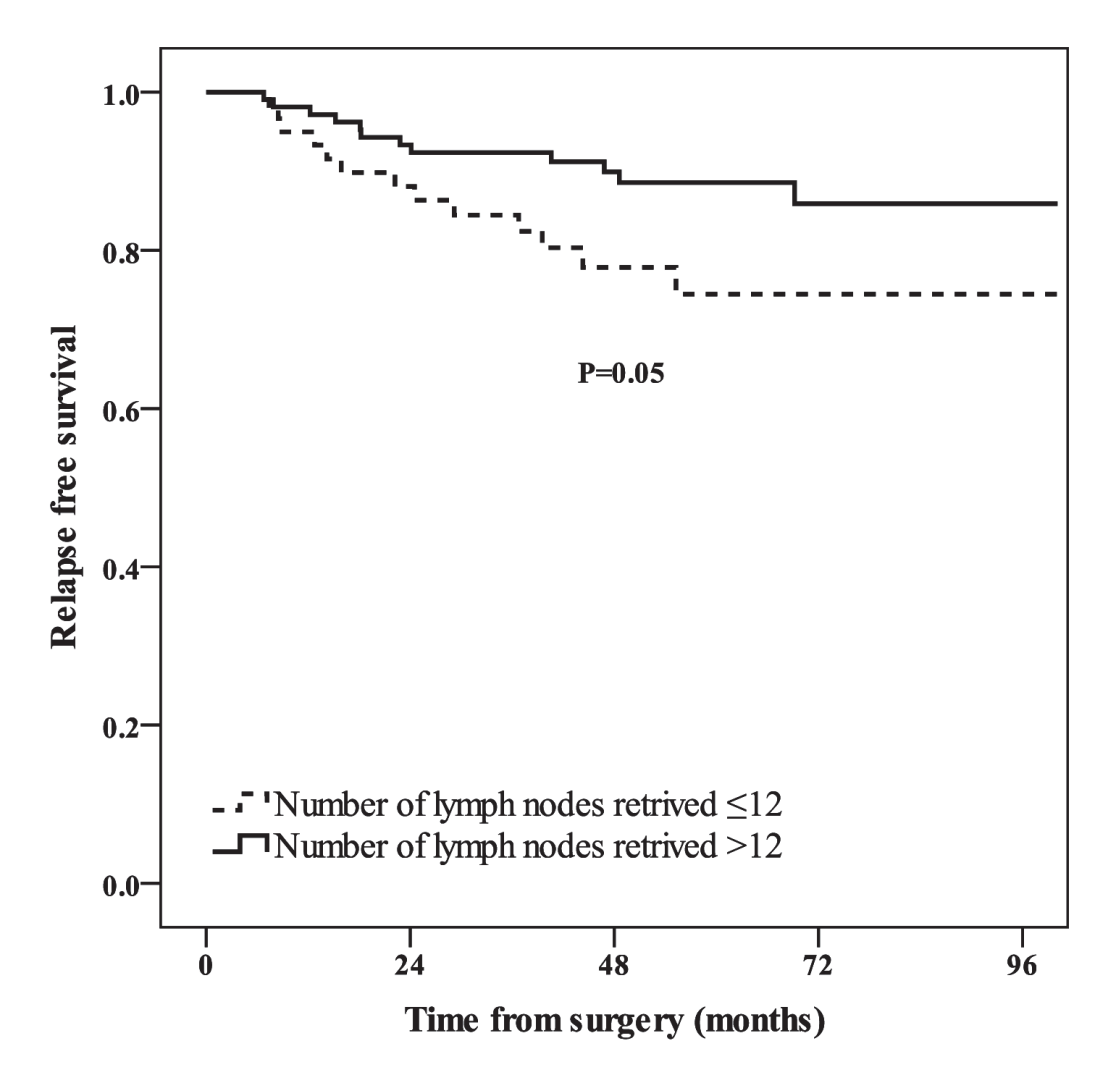
Relapse free survival of patients with number of lymph nodes retrieved≤12 (dashed line), and number of lymph nodes retrieved>12 (solid line).

Inadequate sampling of lymph nodes (≤12) was associated with higher local recurrence rate (P = 0.03, Fig. 2). The relationship between number of lymph nodes retrieved and RFS was marginally significant (P = 0.05, Fig. 3). The 3-year RFS were 84.5% (95% CI: 79.7–89.3) and 92.4% (95% CI: 89.8–95.0) for patients with ≤12 and >12 lymph node sampling, respectively.

## Discussion

In the current study, we identify a group of locally advanced rectal cancer with low risk of local recurrence by tumor location, preoperative MRI assessment of CRM, and TNM staging. For T3N0 rectal cancer with tumor located 5 cm above the anal verge, and without CRM involvement, the local recurrence rate is only 3.4%, which might be low enough to preclude these patients from neoadjuvant CRT.

Local recurrence was once one of the most common types of treatment failure in locally advanced rectal cancer before the standardization of TME surgery [14] and application of neoadjuvant CRT [15]. Although neoadjuvant CRT significantly decreased the risk of local recurrence in locally advanced rectal cancer, the benefit in local control could not be translated into survival benefit [10,16]. Meanwhile, the absolute decrease of local recurrence is moderate, raising the question whether we are over-treating these patients. In a Dutch trial [17], 10-year local recurrence was reduced from 11% to 5% by preoperative short-term radiotherapy. In other words, only 6% of patients benefited from neoadjuvant radiotherapy, meaning that the receipt of radiotherapy by all patients would have resulted in over treatment in 94% of patients. Meanwhile, radiotherapy or CRT inevitably brings short-term and long-term side effects, including hematologic effects, dermatologic effects, strictures at anastomotic site [15], late small bowel obstruction [18], and a substantial increase in bowel frequency and incontinence [19], which further questions the unselective application of neoadjuvant CRT to all locally advanced rectal cancer. As a result, it is critical to identify those patients with low risk of local recurrence and preclude them from the unnecessary neoadjuvant CRT.

A number of studies have demonstrated that patients underwent resection of pT3N0 rectal cancer experience a low rate of local failure after surgery alone. In a group of 108 T3N0 rectal cancer patients treated by TME without adjuvant therapy, Picon et al reported that the 5-year local recurrence rate was 8% [5]. Nissan et al reported a local recurrence rate of 4.1% in a group of patients with pT3N0 treated by radical surgery alone [4]. Similar result was also obtained by Enker showing a local failure of 4% [3]. These studies suggested that neoadjuvant CRT may be excessive for patients with T3N0 rectal cancer. However, not all patients with T3N0 can be spared from neoadjuvant CRT. Location of tumor and CRM status are also closely associated with risk of local recurrence. As reflected by the ESMO guideline for treatment of rectal cancer, a flexible strategy on application of neoadjuvant radiotherapy/CRT is recommended basing on not only clinical staging, but also location of tumor and risk of CRM involvement.

In current study, patients with lower rectal cancer (<5cm from anal verge) were excluded from the study for several concerns. First of all, tumor location is an important indicator for neoadjuvant CRT. It is well accepted that lower locating tumor is associated with higher risk of local recurrence [6]. Meanwhile, tumor involving anterior wall in lower rectum is also linked with higher local recurrence. As a result, neoadjuvant radiotherapy/CRT is recommended even when the staging is only T2 regardless of N staging if the tumor involves anterior wall in low rectum in ESMO guideline for the treatment of rectal cancer [9]. Consequently, the treatment strategy for lower rectal cancer is more complicated and should not be treated equally as the mid and upper rectal cancer. Second, previous studies [20, 21] showed that neoadjuvant CRT may facilitate sphincter-sparing surgery for lower rectal cancer. As a result, sphincter-sparing effect brought by neoadjuvant CRT should also be taken into consideration.

Similarly, pre-operative CRM (or mesorectal fascia) involvement is another critical factor that should be taken into consideration when identifying patients with low risk of recurrence. CRM is a well-established predictor of local recurrence [8]. Studies showed that CRM can be predicted by MRI with high accuracy and consistency [22, 23], allowing preoperative identification of patients at risk of recurrence who will benefit from neoadjuvant CRT, or patients at low risk of recurrence who might be spared from neoadjuvant CRT. In a study of 152 patients with rectal cancer clinically staged as T3 or T2N+, CRM at preoperative staging was the only independent preoperative factor that predicted a higher risk for local recurrence. The 5-year local recurrence rate for patients with a free preoperative CRM was 5.4% [2]. The most recent ESMO guideline of treatment for rectal cancer also emphasizes the sub-category of T3 tumor by MRI. As a result, sub-category of T3N0 tumor by MRI should be considered when identifying patients with low risk of recurrence.

Of note, in the current study, we also found that inadequate sampling of lymph nodes was associated with higher risk of local recurrence. The finding is consistent with previous reports which demonstrated that inadequate sampling of lymph nodes was associated with adverse outcome [24]. This could be explained by incorrect staging caused by inadequate lymph node sampling and the low quality of surgery. In this study, only number of lymph nodes retrieved correlated with local recurrence. In 106 patients with adequate lymph node sampling, there was only one case of local recurrence.

In this study, we defined a sub-group of locally advanced rectal cancer with low risk of local recurrence. To our knowledge, this is the first study to stratify the risk of local recurrence by a multi-factor model consisting of pre-operative CRM status, tumor location, and TNM staging. The relatively low incidence of local recurrence argues against the routine application of neoadjuvant CRT for this population.

The current study is subjective to several limitations. First, it is a retrospective analysis and was therefore limited by the bias inherent in this type of analysis. Second, it included patients with pathologic confirmed stage; whether patients with clinical staging of T3N0 have the same outcome depends on the accuracy of preoperative staging.

## Conclusion

In conclusion, our data suggest that local recurrence rate after TME for upper and middle T3N0 rectal cancer with preoperative CRM>1mm is low and surgery alone may be enough for this group of patients. Large prospective randomized controlled trials should be performed to further investigate this problem.
